# Synchrony 2022: Roundtable Discussion on the Pathways and the Challenges of Getting Medications FDA Approved

**DOI:** 10.3390/jpm13050779

**Published:** 2023-04-30

**Authors:** Gabriel Belfort, Heer Nanda, John C. Slattery, Joanna Sambor, John Rodakis, Richard E. Frye

**Affiliations:** 1 Axial Therapeutics, Woburn, MA 01801, USA; gabriel@axialtx.com; 2 University of California, Berkeley, CA 94720, USA; heer.nanda@berkeley.edu; 3 BioROSA Technologies, Inc., Watertown, MA 02472, USA; jslattery@biorosa.com; 4 MapLight Therapeutics, Redwood City, CA 94063, USA; jsambor@maplightrx.com; 5 N of One: Autism Research Foundation, Dallas, TX 75206, USA; jrodakis@nofone.org; 6 Autism Discovery and Treatment Foundation, Phoenix, AZ 85050, USA

The BRAIN Foundation (Pleasanton, CA, USA) hosted a medicine conference, Synchrony 2022, for research into treatments to benefit individuals with neurodevelopmental disorders (NDDs), including autism spectrum disorders (ASD). One of the four roundtable discussions focused on the challenges surrounding FDA approval for medications to treat NDD and ASD. Gabriel Belfort (M.D., Ph.D), Senior Vice President at Axial Therapeutics (Woburn, MA, USA), chaired the roundtable which also included John C. Slattery (CEO at BioROSA Technologies, Inc., Watertown, MA, USA), Joanna Sambor (Employee at MapLight Therapeutics, Redwood City, CA, USA), John Rodakis (Founder and President at N of One: Autism Research Foundation, Dallas, TX, USA) and others.

The challenges of designing clinical trials and selecting appropriate outcome measures that meet the FDA’s criteria for the approval of new drugs and devices were the main point of discussion. The discussion drew from the collective experience of the participants who had a diverse background in ASD clinical development and regulatory affairs from academia, biopharma and non-profit entities.

The panel identified three major challenges facing those seeking FDA approval for novel treatments:

- Despite a very wide variety of Clinical Outcome Assessment (COA) tools, limited psychometric data exist on which tools are most sensitive to short-term changes in ASD core and ancillary symptomology.
- It is uncertain what the FDA would accept as a clinical trial endpoint for ASD beyond irritability.
- At present, there are no validated biomarkers to aid and expedite the treatment development process for ASD. It was acknowledged that a number of promising biomarkers are under development, such as eye-tracking and various blood-based tests. However, there was agreement that while robust biomarkers would eventually be developed, they are likely years away.

Combing the first two items described above, there was a consensus that one of the most critical, near-term problems for the developers of novel therapeutic approaches is the identification of instruments that both measure changes in ASD domains and are acceptable to regulators.

Several suggestions emerged to address this gap:

- Assembling a working group that, critically, includes the FDA, to understand the strengths and weaknesses of various currently used COA tools and gain feedback from regulators on what they would like to see.
- Since the development, refinement and validation of COA tools require substantial investments, which are often out-of-budget for early-stage biotech companies, the panel proposed advocating for legislation that provides incentives for the development of suitable endpoints (similar to how the Orphan Drug Act (ODA) provides financial incentives to encourage the development of drugs for rare diseases).
- The creation of a Wiki-type repository of endpoints to facilitate the pre-competitive aggregation of trial data toward collectively elucidating the psychometric properties of each instrument.

In discussing COA tools, the panel discussed the relative strengths of caregiver- versus investigator-reported outcomes. The panel agreed that parents and clinicians have complementary roles in the assessment of participants. While parents (and other caregivers) were identified as superior for determining the individual changes in their child during the treatment period, clinicians have a key role in assessing the magnitude of the changing symptoms of a particular participant in relation to the broader group of participants with ASD in the trial.

In the future, the panel is hoping to assemble a working group to discuss these challenges with the FDA and to host more roundtable discussions to elucidate new insight and ideas.

